# Supplementary material for: Does IPV Boost Intestinal Immunity among Children under Five Years of Age? An Experience from Pakistan
Source: Vaccines (Basel). 2023 Sep 1;11(9):1444. doi: 10.3390/vaccines11091444 (PMC10534550; doi:10.3390/vaccines11091444)
Supplement: Supplementary file 1 [file vaccines-11-01444-s001.zip › vaccines-2494912-supplementary.pdf]

Table S1: Serum Samples collected at baseline, V1 and V2, Overall and site wise

	**Baseline**	**Visit 1 (V1)**	**Visit 2 (V2)**
Overall	5972	4895	4552
Karachi	1788	1565	1132
Bajaur	2001	1542	1349
Kashmore	2183	1806	1423

Table S2: Stool Samples collected at Day 0, Day 7 and Day 21, overall and site wise

	**Day 0**	**Day 7**	**Day 21**
Overall	4210	4084	4185
Karachi	1324	1143	1219
Bajaur	1371	1134	1191
Kashmore	1515	1807	1748
